# Supplementary material for: Nurse’s perceptions of support for sexual and reproductive issues in adolescents and young adults with cancer
Source: PLoS One. 2022 Jun 8;17(6):e0265830. doi: 10.1371/journal.pone.0265830 (PMC9176807; doi:10.1371/journal.pone.0265830)
Supplement: S1 File — (PDF) [file pone.0265830.s001.pdf]

Survey for Nurses

Adolescents and Young Adults with Cancer

Table of Content

I. Demographics ········ 1

II. Care difficulties for AYA cancer patients/survivors ········ 3

III. Perceived needs of patients/survivors in daily life ········ 8

IV. Support for sexual and reproductive issues ········ 11

V. End-of-life care ········ 13

VI. Characteristics of AYA patients/survivors whom nurses felt care difficulties ········ 16

VII. Facilitating/disturbing factors for the quality of care for AYAs ········ 18

Ⅰ.Demographics

1. **Institution**

   ①University hospital ②Cancer hospital ③Children hospital ④General hospital

   ⑤Clinic ⑥Others：

2. **Facility certification**

   ①Designated cancer hospitals ②Core hospital for pediatric cancer treatment

   ③Others：

3. **Prefecture**：

4. **Number of beds in the facility**

   ①Less than 200 ②200–399 ③400–599 ④600–799 ⑤More than 800

5. **Affiliated department**

   ①Pediatrics ②Pediatric surgery ③Pediatric hematology/oncology

   ④Hematology/oncology ⑤Internal medicine ⑥Surgery ⑦Neurosurgery

   ⑧Urology ⑨Gynecology ⑩Breast department ⑪Endocrinology

   ⑫Orthopedics ⑬Radiology ⑭Others：

6. **Department**

   ①Ward ②Outpatient ③Ward/outpatient ④Others：

7. **Manager**・・・①No ②Yes (Go to 7-1)

   7-1.

   ①Head nurse ②Deputy-head nurse ③Others：

8. **Certified or specialized nurses**・・・①No ②Yes (Go to 8-1)

   8-1

   ①Certified nurse specialist ②Certified nurse

9. **Total years for nursing**

   ①Less than 1year ②1–3 years ③4–6 years ④7–9 years ⑤More than 10 years

10. **Total years for oncology nursing**

    ①Less than 1year ②1–3 years ③4–6 years ④7–9 years ⑤More than 10 years

11. Cancer-related academic society

①Society of Cancer Nursing ②Society Clinical Oncology ③Society of Medical Oncology

④Society of Palliative Medicine ⑤Society for Radiation Oncology ⑥Radiological Society

⑦Breast Cancer Society ⑧Society of Pediatric Oncology ⑨Psycho-Oncology Society

⑩Others：

Ⅳ. Support for sexual and reproductive issues

1. Do you think it is important to explain to adolescent patients with cancer (age, 15–19 years) the information regarding their sexual and reproductive functions?

①Very important ②Important ③Not very important ④Not important

2. Do you think it is important to explain to young adult patients with cancer (age, 20–39 years) the information regarding their sexual and reproductive functions?

①Very important ②Important ③Not very important ④Not important

3. Do you think that adolescent patients with cancer (age, 15–19 years) are sufficiently debriefed with necessary information regarding their sexual and reproductive functions?

①Sufficient ②Moderately sufficient ③Moderately insufficient ④Insufficient
⑤I don’t know

4. Do you think that young adult patients with cancer (age, 20–39 years) are sufficiently debriefed with the necessary information regarding their sexual and reproductive functions?

①Sufficient ②Moderately sufficient ③Moderately insufficient ④Insufficient
⑤I don’t know

5. Do you think that examinations and care are conducted sufficiently taking sexuality into consideration?

①Sufficient ②Moderately sufficient ③Moderately insufficient ④Insufficient
⑤I don’t know

6. Do you think that a sufficient support system for sexual and reproductive functions is in place?

①Sufficient ②Moderately sufficient ③Moderately insufficient ④Insufficient
⑤I don’t know

7. Support for sexual and reproductive issues

①Referral to other professionals ②Emotional support
③Consultation on fertility preservation ④Consultation on sexual behavior
⑤Consultation on sexuality ⑥Introduction of patient association
⑦Information provided by pamphlets ⑧Consultation on treatment costs
⑨Family support ⑩Symptom management
⑪None ⑫Others：

8. Are nurses present when explaining information regarding their sexual and reproductive functions?

①Always present ②Depending on the situation

③Not present ④I don’t know

資格種類

9. What are the future challenges on support for sexual and reproductive issues for AYA?

①Multidisciplinary collaboration ②Education and professionalization of nurses

③Cooperation with other facilities ④Consultation with expert

⑤Workshops or seminars ⑥Development of a guideline

⑦Others：

10. What difficulties did you encounter in supporting sexual and reproductive problems faced by AYA with cancer?

( )
