# Supplementary material for: Nurse’s perceptions of support for sexual and reproductive issues in adolescents and young adults with cancer
Source: PLoS One. 2022 Jun 8;17(6):e0265830. doi: 10.1371/journal.pone.0265830 (PMC9176807; doi:10.1371/journal.pone.0265830)
Supplement: S2 File — (PDF) [file pone.0265830.s002.pdf]

# 思春期・若年成人がん患者・サバイバーに関する
# 看護師調査

本調査は思春期・若年成人がん患者・サバイバーに関する看護の実態と認識について調査するものです。
本調査は思春期・若年世代がん医療の包括的実態調査（研究代表者　名古屋医療センター臨床研究センター　堀部敬三）の一部です。

**目次**

Ⅰ．属性・・・・・・・・・・・・・・・・・・・・・・・・・・・・・・・・・・・・・・・・・・・・・・・・・・・・・・・・・・・・１

Ⅱ．思春期・若年成人がん患者サバイバーへのケア困難感について・・・・・・・・・・・・・・・・・・・・・・3

Ⅲ．思春期・若年成人がん患者サバイバーのニーズについて・・・・・・・・・・・・・・・・・・・・・・・・・・・・8

Ⅳ．思春期・若年成人がん患者・サバイバーの性に関する支援について・・・・・・・・・・・・・・・・・・11

Ⅴ．思春期・若年成人がん患者の緩和ケア・ターミナルケアについて・・・・・・・・・・・・・・・・・・・・13

Ⅵ．困難事例について・・・・・・・・・・・・・・・・・・・・・・・・・・・・・・・・・・・・・・・・・・・・・・・・・・・・・・16

Ⅶ．思春期・若年成人世代の看護の質向上に関する促進・阻害要因について・・・・・・・・・・・18

**Ⅰ．貴方の所属する施設特性についてお答えください。**

**1．設置主体について、あてはまるものに○をつけてください。**

①大学病院　②がん専門病院　③小児専門病院　④総合病院　⑤診療所

⑥その他（　　　　　　　　　　）

**2．がん診療に関する施設認定について、あてはまるものに○をつけてください。**

①がん診療連携拠点病院　②小児がん拠点病院　③その他（　　　　　　　　　）

**3．貴施設の都道府県についてお答えください。**（　　　　　　　　）都道府県

**4．貴施設の病床数について、あてはまるものに○をつけてください。**

①200 床未満　②200-399 床　③400-599 床　④600-799 床　⑤800 床以上

**5．あなたの所属する診療科について、あてはまるものに○をつけ、（）内に記入してください。**

①小児科　②小児外科　③血液･腫瘍科（小児）　④血液･腫瘍科（成人）

⑤内科（　　　　　　）　⑥外科（　　　　　）⑦脳外科　⑧泌尿器科

⑨婦人科　⑩乳腺科　⑪内分泌科　⑫整形外科　⑬放射線科

⑭その他（　　　　　　　　　　　　　　）

**6．あなたの所属する部署について、あてはまるものに○をつけてください。**

①病棟　②外来　③病棟･外来　④その他（　　　　　　　　　　　）

**7.あなたは管理職ですか？･･･**①いいえ　②はい

7-1．②はいの方のみ　あてはまるものに○を付けてください。

①師長（病棟や看護単位の管理職）　②副師長（①の補佐）

③その他(　　　　　　　　　　　　　　　　　　　　　　　　　　　　　　　)

**8．あなたは認定あるいは専門看護師ですか？･･･**①いいえ　②はい

8-1．②はいの方のみ　あてはまるものに○をつけ専門分野をご記入下さい。

①専門看護師　②認定看護師　　分野名：【　　　　　　　　　　　　　　　　】

**9．看護師としての経験年数について、あてはまるものに○をつけてください。**

①1 年未満　②1-3 年　③4-6 年　④7-9 年　⑤10 年目以上

**10．がん看護の経験年数について、あてはまるものに○をつけてください。**

①1 年未満　②1-3 年　③4-6 年　④7-9 年　⑤10 年目以上

**11．あなたはがん関連の学会に所属していますか？あてはまるものに○をつけてください。**

①日本がん看護学会　②日本癌治療学会　③日本臨床腫瘍学会　④日本緩和医療学会

⑤日本放射線腫瘍学会　⑥日本医学放射線学会　⑦日本乳がん学会　⑧日本小児がん看護学会

⑨日本サイコオンコロジー学会　⑩その他(　　　　　　　　　　　　　　　　　　　　　　)

**Ⅳ. 思春期･若年成人がん患者･サバイバーの性に関する支援について**

**1.思春期がん患者（15-19 歳）に悪性腫瘍やその治療が性･生殖機能に与える影響（影響がない場合でも影響がないこと）を説明することは重要であると思いますか。**

①とても重要である　②重要である　③あまり重要ではない　④重要ではない

**2.若年成人がん患者（20-39 歳）に悪性腫瘍やその治療が性･生殖機能に与える影響（影響がない場合でも影響がないこと）を説明することは重要であると思いますか。**

①とても重要である　②重要である　③あまり重要ではない　④重要ではない

**3. 思春期がん患者（15-19 歳）に性･生殖機能に関する説明や情報提供が十分行われていると思いますか。**

①十分行われていると思う　②どちらかといえば行われている　③どちらかといえば行われていない
④行われていない　⑤わからない

**4. 若年成人がん患者（20-39 歳）に性･生殖機能に関する説明や情報提供が十分行われていると思いますか。**

①十分行われていると思う　②どちらかといえば行われている　③どちらかといえば行われていない
④行われていない　⑤わからない

**5. 思春期･若年成人がん患者･サバイバーのセクシュアリティに配慮した診療やケアが行われていると思いますか。**

①十分行われていると思う　②どちらかといえば行われている　③どちらかといえば行われていない
④行われていない　⑤わからない

**6. 思春期･若年成人がん患者･サバイバーの性･生殖機能に関わる支援体制が整っていると思いますか。**

①整っていると思う　②どちらかといえば整っている　③どちらかといえば整っていない　④整っていない

**7.思春期･若年成人がん患者･サバイバーに対し、性･生殖機能に関する支援について、実施しているもの全てに○をしてください。**

①専門医･カウンセラー･治療機関への紹介　②情緒心理面への支援
③妊孕性温存に関する相談･支援　④性生活に関する相談
⑤セクシャリティに関する相談　⑥患者会の紹介
⑦パンフレット等による情報提供　⑧医療費に関する情報提供･相談窓口の紹介
⑨家族（親･配偶者）への支援　⑩症状マネジメント
⑪実施していない　⑫その他（　　　　　　　　　　）

**8. 性・生殖機能に関する説明に看護師が同席していますか。**

①必ず同席している　②状況に応じて同席している

③同席していない　④わからない

**9.性・生殖機能に関する支援を行うために、今後必要と思われることについて、あてはまるもの全てに○をつけてください。**

①多職種によるチーム医療の充実　②看護師の専門性の向上・教育体制の整備

③他の医療施設との連携　④専門家へのコンサルテーション・スーパーバイズの体制整備

⑤研修会やセミナーの実施　⑥マニュアル・ガイドライン等の充実

⑦その他（　　　　　　　　　　　　　　　　　　　　　　　　　　　　　　）

**10. 思春期・若年成人がん患者・サバイバーへの性に関する支援について、どのようなことに課題や困難を感じますか。お考えを自由にご記入ください。**

（　　　　　　　　　　　　　　　　　　　　　　　　　　　　　　）
