# Supplementary material for: Nurse’s perceptions of support for sexual and reproductive issues in adolescents and young adults with cancer
Source: PLoS One. 2022 Jun 8;17(6):e0265830. doi: 10.1371/journal.pone.0265830 (PMC9176807; doi:10.1371/journal.pone.0265830)
Supplement: Attachment — Submitted filename: Respons to Reviewers.docx [file pone.0265830.s004.docx]

Dear Dr. Emily Chenette

Editor-in-Chief

*PLOS ONE*

Manuscript NO.: PONE-D-21-18301

Title: Nurse’s Perceptions of Support for Sexual and Reproductive Issues in Adolescents and Young Adults with Cancer

Thank you for the opportunity to revise our manuscript. Please see our responses below.

Editor

1. **Comments to author**

Please ensure that your manuscript meets PLOS ONE's style requirements, including those for file naming.

**Response**

We ensured that the style requirements were met and modified the filename.

2. **Comments to author**

Please include additional information regarding the survey or questionnaire used in the study and ensure that you have provided sufficient details that others could replicate the analyses. For instance, if you developed a questionnaire as part of this study and it is not under a copyright more restrictive than CC-BY, please include a copy, in both the original language and English, as Supporting Information. If the original language is written in non-Latin characters, for example Amharic, Chinese, or Korean, please use a file format that ensures these characters are visible.

**Response**

We have added Japanese and English questionnaires to Supporting Information.

3. **Comments to author**

Please state whether you validated the questionnaire prior to testing on study participants. Please provide details regarding the validation group within the methods section.

**Response**

We have stated that we have validated the questionnaire as follows:

*“The researcher’s group consisting of four pediatric cancer nurses, two adult cancer nurses, and two clinical nurses reviewed the questionnaire for surface and content validity.”*

4. **Comments to author**

In your Data Availability statement, you have not specified where the minimal data set underlying the results described in your manuscript can be found. PLOS defines a study's minimal data set as the underlying data used to reach the conclusions drawn in the manuscript and any additional data required to replicate the reported study findings in their entirety. All PLOS journals require that the minimal data set be made fully available. For more information about our data policy, please see <http://journals.plos.org/plosone/s/data-availability>

**Response**

We have added the dataset to Supporting Information.

5. **Comments to author**

Please review your reference list to ensure that it is complete and correct. If you have cited papers that have been retracted, please include the rationale for doing so in the manuscript text, or remove these references and replace them with relevant current references. Any changes to the reference list should be mentioned in the rebuttal letter that accompanies your revised manuscript. If you need to cite a retracted article, indicate the article’s retracted status in the References list and also include a citation and full reference for the retraction notice.
**Response**

We have revised the reference list and provided a rebuttal letter.

Reviewer

We wish to express our sincere appreciation to the reviewers for their insightful comments on our manuscript. As per the reviewers’ suggestions, we have revised the manuscript, and our responses to the reviewers’ comments are as follows:

Reviewer #1:

1. **Comments to author**

some references need to be updated; e.g., King L, Quinn GP, Vadaparampil ST, Gwede CK, Miree CA, Wilson C, et al. Oncology nurses’ perceptions of barriers to discussion of fertility preservation with patients with cancer. Clin J Oncol Nurs. 2008; 12(3): 467-476.

**Response**

Thank you for pointing this out. We have updated reference number14.

2. **Comments to author**

line 68: “The survey period was from June to August 2016” – Authors can　clarify/explicit the reason why the decided to delay the publication.

**Response**

This study was part of a large survey on physicians, nurses, and patients across Japan in 2016, and after analyzing several surveys, we found it useful to focus and report on nurse’s perception of support for sexual and reproductive issues. Therefore, we decided to submit this manuscript. We have stated that this survey was part of a large study as follows:

*“This study was part of a large survey across Japan. The questionnaire had six sections: a) care difficulties for AYA cancer patients/survivors, b) perceived needs of patients/survivors in daily care, c) support for sexual and reproductive issues, d) end-of-life care, e) characteristics of AYA patients/survivors whom nurses felt care difficulties, and f) facilitating/disturbing factors for the quality of care for AYAs. In this study, we used the questionnaire section ‘support for sexual and reproductive issues’.”*

3. **Comments to author**

lines 197-198: “The low effective response rate in this study was a combination of missing answers and the considerable number of nurses who answered “I don’t know,” indicating that nurses themselves are not sufficiently aware of the current situation”. This statement sounds as an inference or a personal authors’ deduction and needs better explanation.

**Response**

Thank you for this suggestion. We agree with the reviewer and have removed the sentence because the low effective response does not indicate that the nurses are not sufficiently aware of the current situation.

4. **Comments to author**

line 70 “measurements”: nurses’ perception investigation could have been more specific, using one or more standardized questionnaire in association with the four questions.

**Response**

Thank you for this suggestion. This was the first exploratory study involving a multidisciplinary team in Japan, and we focused on the background and current status of the subjects. The survey questionnaire consisted of six sections and had many questions, so we did not use the standardized questionnaire for practical reasons. We would like challenge this with further research.

5. **Comments to author**

a comparison with other researches would have been appreciated, even if there are few studies on this topic. It could be interesting a comparison with nurses’ perception of support for sexual and reproductive issues in a different age, or for a different medical problem, or related to other form of support.

**Response**

Thank you for this suggestion. We have added the following text to the Discussion section to compare this study to previous studies investigating the perceptions of healthcare providers in the long-term care of adult cancer survivors:

*“Similarly, in the services of adult cancer survivors, the smallest number of practitioners felt they had skills to manage* *sexual and reproductive issues. Nevertheless, training requirements were not highly prioritized［22］.”*

Reviewer #2:

1. **Comments to author**

This articles does have grammatical errors. In addition, statements at times are written in a very definitive manner, when not appropriate. The authors would benefit from having someone with a more nuanced understanding of the English language review and correct the paper. Please see 2 examples below.

“In addition, cancer treatments, such as chemotherapy and radiation, cause fertility and sexual dysfunction.” The more appropriate sentence would be “In addition, cancer treatments such as chemotherapy and radiation, may cause fertility and sexual dysfunction.” Not all chemotherapy and radiation treatments cause problems with fertility and sexual dysfunction.
Another example- “Receiving a diagnosis and the subsequent treatment of cancer during this life stage inevitably disrupts their outlook regarding a promising future.” This statement is very definitive and assumes the future is not promising. While getting diagnosed and treated for cancer will cause some disruption in the life of AYA’s, their future can still be promising and some patients even report psychological benefits from the cancer experience. Perhaps a preferable way to phrase the statement- “Receiving a diagnosis and the subsequent treatment of cancer during this life stage inevitably disrupts their life outlook.”
These are just 2 examples found throughout the article. This reviewer suggests having the authors review for grammatical errors and correct. In addition, this reviewer suggests reviewing whether definitive statements have research data to back up the conclusions stated.

**Response**

Thank you for this suggestion. We have revised two sentences based on your suggestion. Moreover, we have proofread the manuscript again.

2. **Comments to author**

The organization of the “free descriptions” was confusing as there are too many codes and categories. The authors may want to consider a more succinct way of organizing and reporting the qualitative information.

**Response**

Thank you for this suggestion. We have simplified the code because there are several qualitative codes. Moreover, we have shown only one.

All abovementioned changes have been reflected in the manuscript in red text.

We hope you find the revised manuscript acceptable for publication. Thank you once again for the consideration.

Yours Sincerely,

Akiko Tomioka, RN, PhD

Professor, Faculty of Healthcare, Tokyo Healthcare University, Tokyo, Japan

Department of Pediatric Nursing

Address: 4-1-17 Higashi-gotanda, Shinagawa-ku, Tokyo 141-8648, Japan

Tel: +81-3-5421-7655

E-mail: [a-tomioka@thcu.ac.jp](mailto:a-tomioka@thcu.ac.jp)
